# The Pseudomonas aeruginosa Type III Secretion System Exoenzyme Effector ExoU Induces Mitochondrial Damage in a Murine Bone Marrow-Derived Macrophage Infection Model

**DOI:** 10.1128/iai.00470-21

**Published:** 2022-03-17

**Authors:** Kierra S. Hardy, Amanda N. Tuckey, Nicole A. Housley, Joel Andrews, Mita Patel, Abu-Bakr Al-Mehdi, Robert A. Barrington, Suzanne L. Cassel, Fayyaz S. Sutterwala, Jonathon P. Audia

**Affiliations:** a Department of Microbiology and Immunology, University of South Alabama College of Medicine, Mobile, Alabama, USA; b Center for Lung Biology, University of South Alabama College of Medicine, Mobile, Alabama, USA; c Mitchell Cancer Institute, University of South Alabama College of Medicine, Mobile, Alabama, USA; d Department of Pharmcology, University of South Alabama College of Medicine, Mobile, Alabama, USA; e Women’s Guild Lung Institute, Department of Medicine, Cedars-Sinai Medical Center, Los Angeles, California, USA; Yale University School of Medicine

**Keywords:** *Pseudomonas aeruginosa*, ExoU, ExoT, NLRC4 inflammasome, caspase-1, mitochondria, pneumonia, sepsis

## Abstract

Pseudomonas aeruginosa is a Gram-negative, opportunistic pathogen that causes nosocomial pneumonia, urinary tract infections, and bacteremia. A hallmark of P. aeruginosa pathogenesis is disruption of host cell function by the type III secretion system (T3SS) and its cognate exoenzyme effectors. The T3SS effector ExoU is phospholipase A_2_ (PLA_2_) that targets the host cell plasmalemmal membrane to induce cytolysis and is an important virulence factor that mediates immune avoidance. In addition, ExoU has been shown to subvert the host inflammatory response in a noncytolytic manner. In primary bone marrow-derived macrophages (BMDMs), P. aeruginosa infection is sensed by the nucleotide-binding domain containing leucine-rich repeats-like receptor 4 (NLRC4) inflammasome, which triggers caspase-1 activation and inflammation. ExoU transiently inhibits NLRC4 inflammasome-mediated activation of caspase-1 and its downstream target, interleukin 1β (IL-1β), to suppress activation of inflammation. In the present study, we sought to identify additional noncytolytic virulence functions for ExoU and discovered an unexpected association between ExoU, host mitochondria, and NLRC4. We show that infection of BMDMs with P. aeruginosa strains expressing ExoU elicited mitochondrial oxidative stress. In addition, mitochondria and mitochondrion-associated membrane fractions enriched from infected cells exhibited evidence of autophagy activation, indicative of damage. The observation that ExoU elicited mitochondrial stress and damage suggested that ExoU may also associate with mitochondria during infection. Indeed, ExoU phospholipase A_2_ enzymatic activity was present in enriched mitochondria and mitochondrion-associated membrane fractions isolated from P. aeruginosa-infected BMDMs. Intriguingly, enriched mitochondria and mitochondrion-associated membrane fractions isolated from infected *Nlrc4* homozygous knockout BMDMs displayed significantly lower levels of ExoU enzyme activity, suggesting that NLRC4 plays a role in the ExoU-mitochondrion association. These observations prompted us to assay enriched mitochondria and mitochondrion-associated membrane fractions for NLRC4, caspase-1, and IL-1β. NLRC4 and pro-caspase-1 were detected in enriched mitochondria and mitochondrion-associated membrane fractions isolated from noninfected BMDMs, and active caspase-1 and active IL-1β were detected in response to P. aeruginosa infection. Interestingly, ExoU inhibited mitochondrion-associated caspase-1 and IL-1β activation. The implications of ExoU-mediated effects on mitochondria and the NLRC4 inflammasome during P. aeruginosa infection are discussed.

## INTRODUCTION

Pseudomonas aeruginosa is a Gram-negative, opportunistic pathogen that is associated with more than 51,000 health care-associated infections annually (1). Ventilator-associated pneumonia (VAP) elicited by P. aeruginosa is a leading cause of acute respiratory distress syndrome (ARDS) and sepsis (2–5). P. aeruginosa is responsible for approximately 40% of deaths of patients with VAP (6). Despite advances in critical care, and early goal-directed antibiotic therapy, pneumonia survivors suffer reduced quality of life, including recurrent hospital readmission and decreased life expectancy. Compounding this issue is the fact that microbes rapidly acquire antibiotic resistance making them difficult, if not impossible, to treat. Due to its high propensity for antibiotic resistance, P. aeruginosa is designated an ESKAPE pathogen (Enterococcus faecium, Staphylococcus aureus, Klebsiella pneumoniae, Acinetobacter baumannii, P. aeruginosa, and Enterobacter species) and is ranked 2nd on the World Health Organization Critical Priority 1 list for new research and drug development (7–10).

Pseudomonas aeruginosa employs a variety of virulence mechanisms to subvert host cellular defenses. The most notorious P. aeruginosa virulence determinant is the type III secretion system (T3SS), a specialized protein injection complex (4, 5, 11). The T3SS injects exoenzyme (Exo) effectors directly into the host cell cytosol, where they become active upon interaction with their cognate eukaryotic protein cofactors (11–16). Together, the T3SS and exoenzyme effectors target and disrupt host immune, epithelial, and endothelial cell function, leading to development of pneumonia, ARDS, and sepsis. There are four well-studied effector proteins: ExoS, ExoT, ExoY, and ExoU (17–20). Of the four effector proteins, ExoU is the most potent inducer of rapid host cell death and dysfunction (19, 21–25). ExoU is present in nearly 30% of P. aeruginosa clinical isolates, and strains encoding ExoU are associated with poor clinical outcomes (2–4, 19, 26–28). ExoU is a ubiquitin-activated phospholipase A_2_ (PLA_2_) that targets the host plasmalemmal membrane to cause rapid lysis (12, 22, 24, 29). ExoU-mediated cell lysis is considered a key virulence determinant that subverts the host immune response to infection. For example, in murine infection models, ExoU targets and kills neutrophils infiltrating the lung as a mechanism of immune avoidance (21, 30).

ExoU has also been shown to play noncytolytic roles in subversion of the host inflammatory response to infection (for a recent review, see reference 31). Innate immune pattern recognition signaling plays a critical role in sensing pathogen- or damage-associated molecular patterns (PAMPs or DAMPs, respectively) (32–34). The P. aeruginosa T3SS needle tip complex and flagellin are PAMPs that specifically activate the intracellular nucleotide-binding domain containing leucine rich repeats-like receptor 4 (NLRC4)/NLR family apoptosis inhibitor proteins (NAIP) inflammasome complex during infection (35–38). Inflammasome activation drives the host inflammatory response through autoproteolysis of pro-caspase-1 and subsequent processing of proinflammatory cytokines, interleukin 1β (IL-1β) and IL-18, and activation of gasdermin D, the executioner of pyroptosis (33, 39–42). ExoU has been previously shown to transiently inhibit NLRC4 inflammasome activation, which suppresses the IL-1β-mediated inflammatory responses to P. aeruginosa infection (43, 44). In addition, ExoU has been shown to disrupt IL-1 family cytokine activation in the lung to augment P. aeruginosa virulence (45) and to exacerbate neutrophil migration into the lung (46). Together, these data suggest that ExoU plays important cytolytic and noncytolytic roles in subverting the host innate immune response to infection. In the present study, we sought to identify additional noncytolytic virulence functions for ExoU. Our data implicate a novel pathogen-host relationship between ExoU, mitochondria, and NLRC4 that potentially functions to subvert the host innate immune response to P. aeruginosa infection.

## RESULTS

### P. aeruginosa ExoU transiently inhibits NLRC4 inflammasome activation during infection of BMDMs.

**FIG 1:**
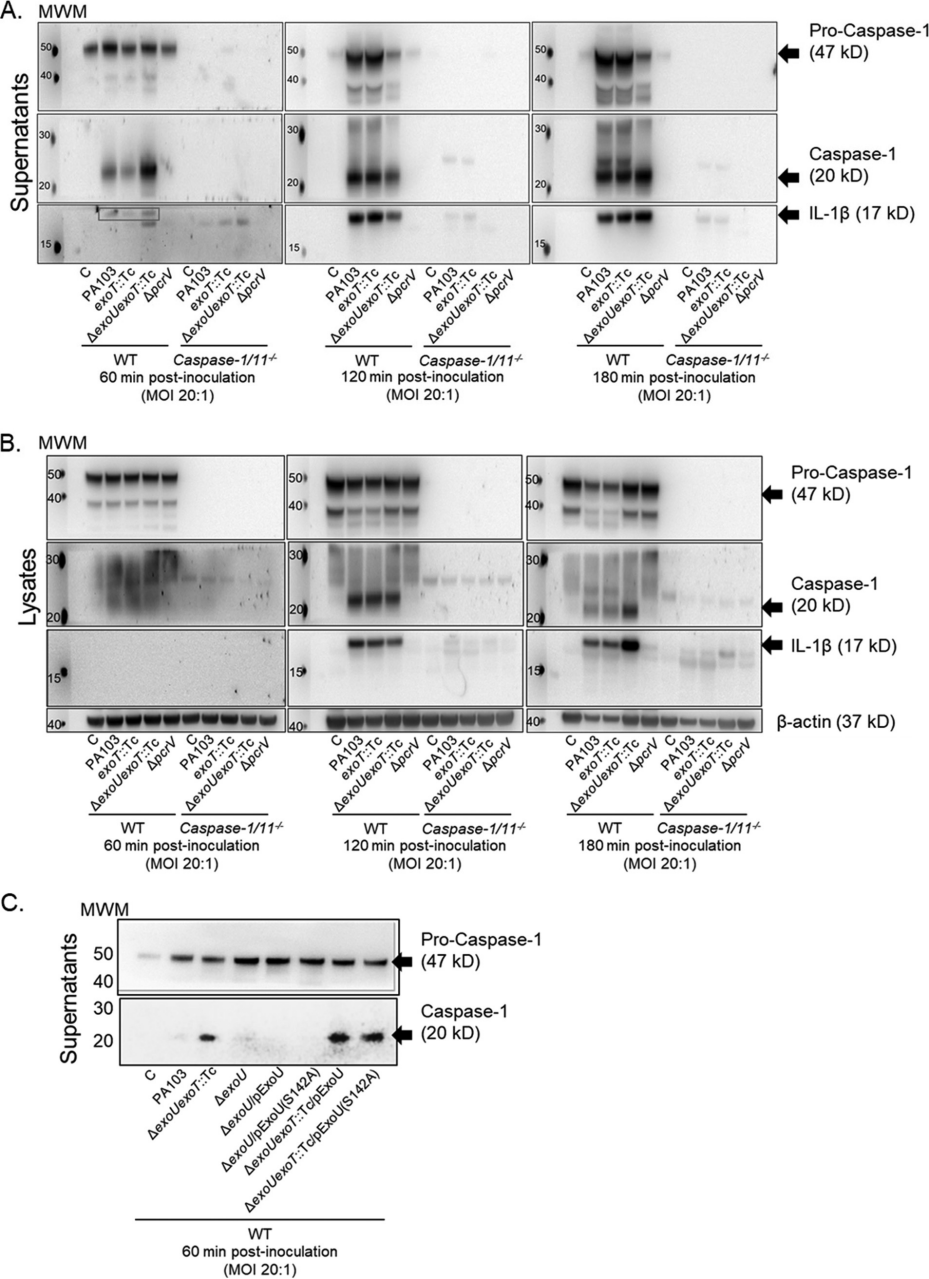
P. aeruginosa T3SS effector ExoU inhibits caspase-1 and IL-1β activation during infection. (A) Wild-type (WT) and *caspase-1/11^−/−^* BMDMs were treated with sterile saline solution (C) or inoculated with P. aeruginosa strains as indicated (also see Table 1). Pro-caspase-1 (47 kDa), active caspase-1 (20 kDa), and active IL-1β (17 kDa; see also boxed section in the 60-min time point blots) were measured over time in TCA-concentrated supernatants by Western blotting. Note that in this and subsequent blots, the lanes immediately to the right of the molecular weight marker (MWM) spots are intentionally empty. (B) Pro-caspase-1, active caspase-1, active IL-1β, and β-actin (loading control) were measured in WT and *caspase-1/11^−/−^* BMDM lysates collected under control or P. aeruginosa inoculation conditions. (C) Pro-caspase-1 and active caspase-1 were measured in WT BMDM TCA-concentrated supernatants from control or P. aeruginosa strains complemented with plasmids expressing either wild-type ExoU or activity-null ExoU (S142A). MOI, multiplicity of infection. All Western blots are representative of 3 independent experiments.

Our studies employed P. aeruginosa wild-type (WT) strain PA103, which expresses the ExoU and ExoT T3SS effectors and lacks motility (19). During infection, the P. aeruginosa T3SS needle tip complex and flagellin are the major PAMPs that activate the NLRC4 inflammasome (35–38). Thus, in our PA103 model system, the T3SS needle tip complex is the primary NLRC4 inflammasome activator. In addition, ExoU in strain PA103 has been previously shown to inhibit caspase-1 activation in murine bone marrow-derived macrophages (BMDMs) and rat lung endothelial cells (43, 44). Based on these previous observations, we first set out to establish an infection model using murine WT and *caspase-1/11^−/−^* BMDMs inoculated with WT PA103, isogenic PA103 mutants encoding ExoU only (*exoT*::Tc) or ExoT only (Δ*exoU*) or lacking both ExoU and ExoT (Δ*exoU exoT*::Tc), or a T3SS mutant (Δ*pcrV*) (genotypes described in Table 1). NLRC4 inflammasome activation was assayed by Western blotting for processed caspase-1 and IL-1β in clarified culture supernatants and cell lysates over time. Note that the model used in this study did not involve priming the macrophages with a Toll-like receptor agonist (therefore, IL-1β levels were low at early time points). As a positive control, BMDMs were inoculated with a mutant PA103 strain expressing a functional T3SS needle tip complex, but lacking ExoU and ExoT, to activate the NLRC4 inflammasome (Δ*exoU exoT*::Tc in Fig. 1A and B) (47). As a negative control, inoculation of BMDMs with a mutant PA103 strain lacking a functional T3SS needle tip complex did not activate NLRC4 inflammasome over the time course tested (Δ*pcrV* in Fig. 1A and B) (48).

**TABLE 1:** Bacterial strains used in this study

Bacterial strain	Genotype	Phenotype	Reference or comment
PA103	Wild type	Wild type	19
PA103 ΔU	Δ*exoU*	Attenuated	19
PA103 ΔT	*exoT*::Tc	Attenuated	19
PA103 ΔUT	Δ*exoU exoT*::Tc	Attenuated	46
PA103 Δ*pcrV*	Δ*pcrV*	Avirulent	47
PA103 ΔU/pExoU	Δ*exoU/*pUCP18-*exoU*	Complemented	Transformant generated for this study
PA103 ΔU/pExoU (S142A)	Δ*exoU/*pUCP18-*exoU*(S142A)	Complemented, ExoU active-site mutant	Transformant generated for this study
PA103 ΔUT/pExoU	Δ*exoU exoT*::Tc*/*pUCP18-*exoU*	Complemented	Transformant generated for this study
PA103 ΔUT/pExoU (S142A)	Δ*exoU exoT*::Tc*/*pUCP18-*exoU* (S142A)	Complemented, ExoU active-site mutant	Transformant generated for this study

As expected, inoculation of BMDMs with P. aeruginosa strains expressing ExoU resulted in transient inhibition of caspase-1 and IL-1β activation, which occurred for the first 60 min postinoculation (Fig. 1A). In cell lysate fractions from BMDMs infected with ExoU-expressing strains, caspase-1 and IL-1β levels were lower at 180 min postinoculation, but this difference was likely due to cell death induced by pyroptosis and/or ExoU PLA_2_ activity (Fig. 1B, compare active caspase-1 and IL-1β levels to the β-actin loading control). As a control for antibody specificity in the Western blot, we tested *caspase-1/11^−/−^* BMDMs, which did not show any pro-caspase-1 and showed substantially diminished levels of IL-1β activation under the conditions tested (Fig. 1A and B). As an additional set of control experiments, we complemented the PA103 Δ*exoU* and Δ*exoU exoT*::Tc mutants with plasmids expressing either wild-type ExoU or activity-null ExoU (S142A) and examined caspase-1 activation at 60 min postinoculation. Interestingly, the PA103 Δ*exoU* mutant alone also elicited inhibition of caspase-1 activation (Fig. 1C). These data suggest that ExoT may also inhibit caspase-1 activation under the culture and infection conditions used in this study. In addition, plasmid-mediated expression of wild-type ExoU did not complement the PA103 Δ*exoU exoT*::Tc mutant phenotype (Fig. 1C), suggesting that overexpression of ExoU may have pleiotropic and/or concentration-dependent effects during P. aeruginosa infection of BMDMs. Finally, we also confirmed the dependence of caspase-1 and IL-1β activation on the NLRC4 inflammasome in this model using *Nlrc4^−/−^* BMDMs (data not shown). These results validated our infection model and indicated that ExoU and ExoT inhibit NLRC4 inflammasome as a noncytolytic mechanism to subvert the host response to infection.

### P. aeruginosa ExoU elicits oxidative stress and mitochondrial damage during infection of BMDMs.

**FIG 2:**
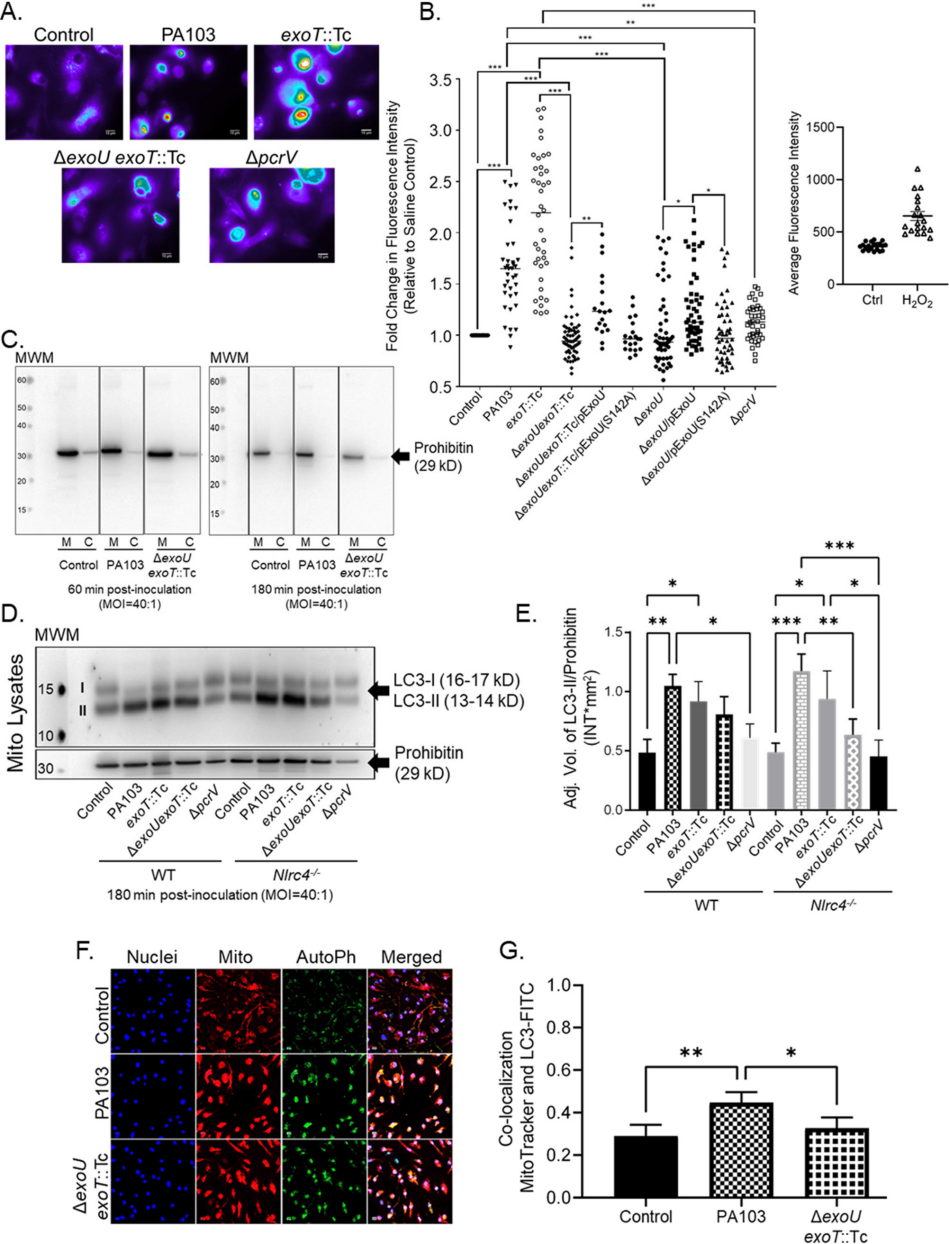
ExoU elicits oxidative stress and induces autophagy at the mitochondria during P. aeruginosa infection. (A) MitoSOX-labeled WT BMDMs were treated with sterile saline (control) or inoculated with P. aeruginosa strain PA103, PA103 *exoT*::Tc, PA103 Δ*exoU exoT*::Tc, or PA103 Δ*pcrV* at an MOI of 20:1. At 180 min postinoculation, images were acquired by fluorescence microscopy. Images are representative of 3 to 7 biological replicates. The scale bar is equal to 10 μm. (B) Average fluorescence intensity of images from panel A, including additional strains as indicated. Data for several of the groups were determined to be non-normally distributed (D’Agostino and Pearson normality test); thus, all groups were compared by one-way ANOVA with Kruskal-Wallis *post hoc* test. All statistical comparison data are reported in Table S1. As a control, WT BMDMs were treated with either cDMEM (Ctrl) or H_2_O_2_ (400 μM) for 180 min. Images were acquired by fluorescence microscopy and intensities determined (2 biological replicates). (C) WT BMDMs treated with sterile saline (control) or inoculated with P. aeruginosa strain PA103 or PA103 Δ*exoU exoT*::Tc for 60 min or 180 min. Enriched mitochondrial (M) and cytosol (C) fractions (2 μg of total protein) were assayed by Western blotting to measure prohibitin as a marker of mitochondrial enrichment (29 kDa). Blots were run on separate gels and images compiled. (D) WT or *Nlrc4^−/−^* BMDMs were treated with sterile saline (control) or inoculated with P. aeruginosa strain PA103, PA103 *exoT*::Tc, PA103 Δ*exoU exoT*::Tc, or PA103 Δ*pcrV*. At 180 min postinoculation, enriched mito-MAM fractions were isolated, and LC3-I, LC3-II, and prohibitin were measured in lysates by Western blotting. (E) Densitometry analysis of blots from panel D showing LC3-II signal normalized to prohibitin. ***, *P* value ≤ 0.0007; **, *P* value ≤ 0.008; *, *P* value ≤ 0.04. All blots and densitometry analyses are representative of 3 independent experiments. (F) Colocalization of LC3 with mitochondria. WT BMDMs were treated with sterile saline (control) or inoculated with P. aeruginosa strain PA103 or PA103 Δ*exoU exoT*::Tc for 180 min at an MOI of 20:1. Images of red (MitoTracker) and green (LC3) fluorescence were acquired by confocal microscopy. Colocalization of LC3 with mitochondria appears as yellow to orange spots. (G) Colocalization analysis using Pearson’s correlation coefficient. **, *P* value = 0.003; *, *P* value = 0.01. Images are representative of 4 independent experiments.

We next sought to determine whether ExoU exerts additional noncytolytic effects on BMDMs during P. aeruginosa infection. Prior evidence suggests that P. aeruginosa induces mitochondrial oxidative stress and damage, but a specific role for ExoU has not been described (49). These observations prompted us to ask whether ExoU elicits oxidative stress and mitochondrial damage in our BMDM infection model. For these experiments, we selected a time point later in the infection to ascertain whether any ExoU-mediated effects occur after the NLRC4 inhibition has been relieved. To this end, we assessed oxidative stress in MitoSOX-labeled BMDMs at 180 min after inoculation with wild-type PA103 and mutant strains (with or without plasmid-mediated complementation) listed in Table 1. Infection with P. aeruginosa strains expressing ExoU elicited elevated levels of oxidative stress compared to that in uninfected controls (Fig. 2A and B). Infection with mutant strains lacking ExoU and ExoT or a functional T3SS elicited only a modest increase in oxidative stress compared to that in uninfected controls (Fig. 2A and B). Intriguingly, plasmid-mediated complementation of mutant strain PA103 Δ*exoU* or PA103 Δ*exoU exoT*::Tc with activity-null ExoU (S142A) did not affect oxidative stress levels during infection (Fig. 2B). Conversely, plasmid-mediated complementation of mutant strain PA103 Δ*exoU* or PA103 Δ*exoU exoT*::Tc with wild-type ExoU resulted in increased levels of oxidative stress (Fig. 2B). Together, these data suggest that ExoU enzymatic activity is required for induction of oxidative stress during P. aeruginosa infection of BMDMs. As a positive control, MitoSOX-labeled BMDMs were treated with H_2_O_2_ (Fig. 2B).

We next examined ExoU-mediated effects on mitochondria by assaying enriched mitochondria and mitochondrion-associated membrane (mito-MAM) fractions for evidence of mitochondrial damage. First, we verified the extent of mito-MAM enrichment in our model using prohibitin, a known mitochondrion-associated control protein (50, 51). As a proof-of-concept experiment, we showed that the levels of prohibitin are substantially enriched in mito-MAM fractions compared to cytosolic fractions isolated from BMDMs under control (uninfected) or infected with wild-type PA103 or the PA103 Δ*exoU exoT*::Tc mutant (Fig. 2C). It is important to note that we also detected various levels of the cytosolic proteins β-actin and GAPDH (glyceraldehyde phosphate dehydrogenase) in the mito-MAM fractions by Western blotting (data not shown). Thus, we refer to the mito-MAM fractions as enriched to acknowledge possible contributions from other cytosolic membrane fractions. To determine the extent of mitochondrial damage, we assayed mito-MAM fractions for microtubule-associated protein light chain 3B (LC3) forms LC3-I and LC3-II as canonical markers of autophagy activation (52). At 180 min postinoculation, P. aeruginosa strains expressing ExoU activated autophagy, as indicated by increased amounts of LC3-II in enriched mito-MAM fractions compared to uninfected controls or the case with infection with the PA103 Δ*pcrV* mutant strain lacking a functional T3SS (Fig. 2D and E). We also observed a difference in autophagy activation between wild-type PA103 and the PA103 Δ*exoU exoT*::Tc mutant strain in mito-MAM fractions enriched from *Nlrc4^−/−^* BMDMs (Fig. 2D and E); however, the biological significance of this difference remains unclear. In addition, we did not observe any significant differences in autophagy activation in enriched mito-MAM fractions isolated at 60 min postinoculation (see Fig. S1A and B in the supplemental material), and analyses of LC3-I and LC3-II in whole-cell extracts did not show differences between any of the conditions or time points tested (Fig. S1C). Together, these data suggest that P. aeruginosa infection may elicit distinct effects on autophagy activation at the mitochondria.

Considering potential issues in resolving different forms of LC3 by Western blotting (53), we augmented our approach using confocal fluorescence microscopy as an independent method to test for autophagy activation at the mitochondria. BMDMs were stained with MitoTracker Red CMXRos, infected for 180 min, and then fixed for immunostaining to assay total LC3. Compared to the uninfected controls, we found significantly higher levels of LC3 and mitochondrial colocalization during P. aeruginosa infection (Fig. 2F and G). Importantly, infection with wild-type PA103 resulted in increased LC3 and mitochondrial colocalization compared to the uninfected control and the PA103 Δ*exoU exoT*::Tc mutant strain (Fig. 2G). As an additional indicator of mitochondrial stress during P. aeruginosa infection, we measured expression of VDAC-1 in mito-MAM fractions enriched from wild-type and *Nlrc4^−/−^* BMDMs. VDAC-1 levels are known to increase during mitochondrial stress and VDAC-1 then forms mitochondrial outer membrane pores (54). We observed a significant increase in VDAC-1 levels between wild-type PA103 and the uninfected control or the PA103 Δ*exoU exoT*::Tc mutant in mito-MAM fractions enriched from wild-type BMDMs (Fig. S1D and E). In mito-MAM fractions enriched from *Nlrc4^−/−^* BMDMs, there was a significant increase in VDAC-1 levels between wild-type PA103 and the PA103 Δ*exoU exoT*::Tc mutant. These findings strongly suggest that ExoU induces mitochondrial oxidative stress and damage during P. aeruginosa infection of BMDMs.

### P. aeruginosa ExoU associates with mitochondria during infection, and the association involves NLRC4.

**FIG 3:**
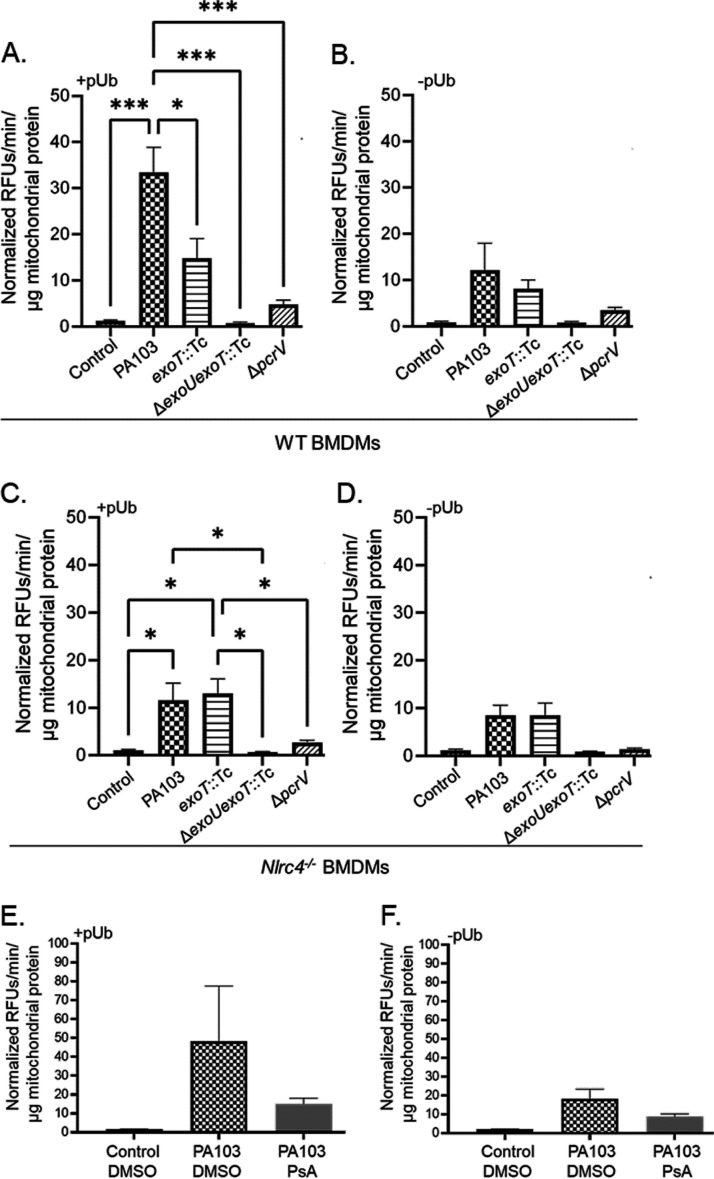
ExoU associates with mitochondria via NLRC4 and ExoT. (A and B) WT BMDMs were treated with sterile saline (control) or inoculated with P. aeruginosa strain PA103, PA103 *exoT*::Tc, PA103 Δ*exoU exoT*::Tc, or PA103 Δ*pcrV* at an MOI of 40:1. At 180 min postinoculation, enriched mito-MAMs were isolated and suspended directly into buffered solution containing the fluorogenic PLA_2_ substrate PED6. Samples were then split into two separate reactions. Panel A shows reactions where pUb was added (0.1 mg/mL; +pUb) to stimulate ExoU PLA_2_ activity in the enriched mito-MAM fractions. Panel B shows control reactions where pUb was not added (–pUb). All raw data (relative fluorescent units [RFUs]) were corrected for background fluorescence, and PLA_2_ activity is expressed as the rate of PED6 hydrolysis (in minutes), normalized to total mitochondrial protein in each reaction (in micrograms). (C and D) *Nlrc4^−/−^* BMDMs were treated and tested for ExoU using the PED6 PLA_2_ assay as described for panels A and B. Panel C shows reactions where pUb was added (0.1 mg/mL; +pUb), and panel D shows control reactions where pUb was not added (–pUb). ***, *P* value ≤ 0.0005; *, *P* value ≤ 0.04. (E and F) WT BMDMs were preincubated with DMSO (vehicle control) or the ExoU-specific inhibitor PsA (50 μM) for 60 min prior to inoculation. Subsequently, cultures were treated with sterile saline (control) or inoculated with P. aeruginosa strain PA103 for 180 min (DMSO or PsA was maintained throughout the time course). Enriched mito-MAM fractions were isolated and tested for ExoU using the PED6 PLA_2_ assay. Panel E shows reactions where pUb was added (0.1 mg/mL), and panel F shows control reactions where pUb was not added. Data in all panels are representative of 3 independent experiments.

Thus far, the data raise the intriguing prospect that ExoU associates with mitochondria to induce oxidative stress and damage during P. aeruginosa infection of BMDMs. To measure ExoU in enriched mito-MAM fractions, we adapted a highly sensitive fluorogenic PLA_2_ activity assay (Fig. 3). For these experiments, we first enriched mito-MAM fractions from control or infected BMDMs at 180 min postinoculation. Subsequently, the enriched mito-MAM fractions were suspended directly into a buffered solution containing a fluorogenic phospholipid substrate, PED6 [*N*-((6-(2,4-dinitrophenyl)amino)hexanoyl)-2-(4,4-difluoro-5,7-dimethyl-4-bora-3a,4a-diaza-*s*-indacene-3-pentanoyl)-1-hexadecanoyl-*sn*-glycero-3-phosphoethanolamine], to facilitate detection of any ExoU PLA_2_ activity that was coenriched. The key to this assay is that ExoU PLA_2_ activity can be readily distinguished from host PLA_2_ activity because ExoU is stimulated by its eukaryotic cofactor, polyubiquitin (pUb). Thus, for these assays, each reaction was split in two and incubated with and without addition of recombinant pUb. The rate of PED6 hydrolysis was expressed as relative fluorescent units (RFUs) over time, normalized to total mitochondrial protein added to the reaction.

To verify the assay, we first confirmed that pUb-stimulated PLA_2_ activity was highest in enriched mito-MAM fractions isolated from BMDMs infected with wild-type PA103 (compare Fig. 3A [with pUb] and Fig. 3B [without pUb]). As expected, the uninfected control and the negative controls, the PA103 Δ*exoU exoT*::Tc and PA103 Δ*pcrV* mutant strains, did not elicit any pUb-stimulated PLA_2_ activity (Fig. 3A and B). In order to estimate a relative amount of ExoU enzyme activity in the enriched mito-MAM fractions, we compared values for extracts to the activity measured using known amounts of purified recombinant ExoU with an N-terminal His_10_ tag (N-His_10_-ExoU) (15). In enriched mito-MAM fractions isolated from BMDMs infected with the wild-type PA103 strain, we detected an average ExoU PLA_2_ activity equivalent to ∼0.11 μg/reaction as interpolated from a standard curve using purified recombinant N-His_10_-ExoU (data not shown).

Unexpectedly, pUb-stimulated ExoU PLA_2_ activity was reduced in enriched mito-MAM fractions isolated from BMDMs infected with a PA103 *exoT*::Tc mutant strain lacking ExoT compared to wild-type PA103 (Fig. 3A). Furthermore, pUb-stimulated ExoU PLA_2_ activity was also reduced in mito-MAM fractions isolated from *Nlrc4^−/−^* BMDMs infected with wild-type PA103 (compare Fig. 3A to C and Fig. 3D). Interestingly, the pUb-stimulated ExoU PLA_2_ activities were comparable between wild-type and *caspase-1/11^−/−^* BMDMs infected with strains expressing ExoU, suggesting that pro-caspase-1 or active caspase-1 is not involved in the ExoU-mitochondrion association (data not shown).

We next asked whether ExoU PLA_2_ activity was required for the observed ExoU-mitochondrion association. For these experiments, we used pseudolipasin A (PsA), a specific inhibitor of ExoU PLA_2_ activity that does not inhibit host PLA_2_ (55). The vehicle control (dimethyl sulfoxide [DMSO]) or PsA (50 μM) was added 60 min prior to inoculation and maintained throughout the time course. Enriched mito-MAM fractions were then isolated and added to the PED6 PLA_2_ assay. Compared to the case with an uninfected control or a vehicle control (DMSO), the addition of PsA during the infection reduced the amount of ExoU PLA_2_ activity detected in enriched mito-MAM fractions, although the data did not reach statistical significance (Fig. 3E and F). These data suggest that ExoU associates with mitochondria and that maximal association involves NLRC4 or ExoT and may also require ExoU PLA_2_ enzyme activity.

### P. aeruginosa ExoU inhibits caspase-1 activation at the mitochondria.

**FIG 4:**
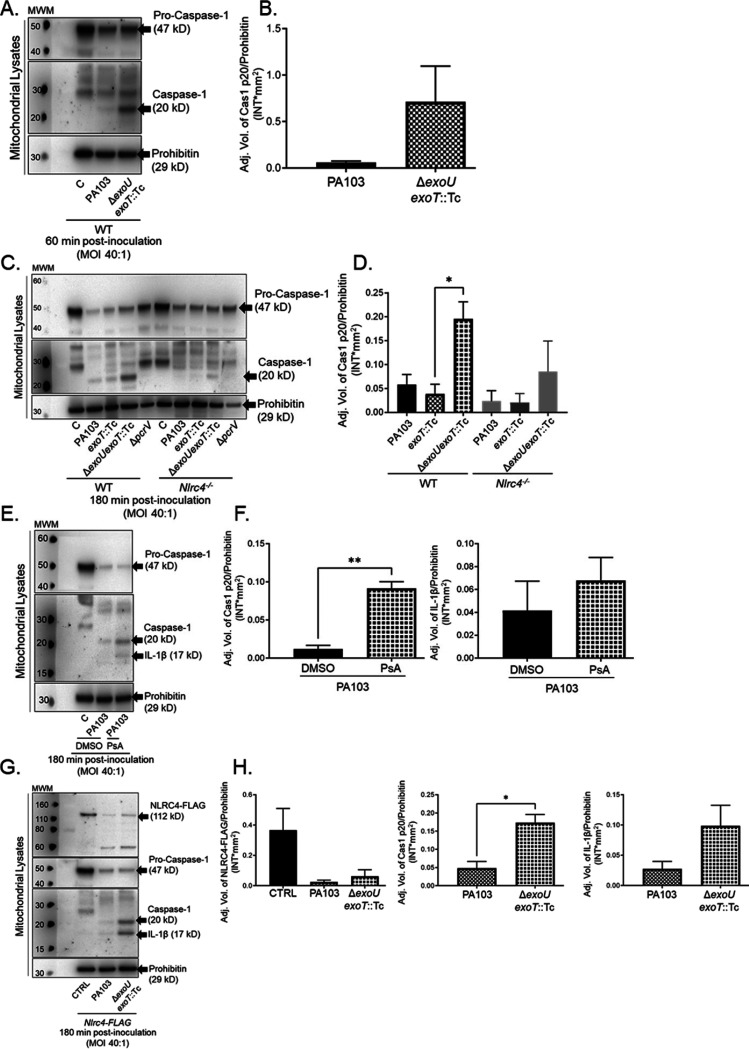
ExoU inhibits caspase-1 activation at the mitochondria. (A) WT BMDMs were treated with sterile saline (C) or inoculated with P. aeruginosa strains PA103 or PA103 Δ*exoU exoT*::Tc. At 60 min postinoculation, enriched mito-MAM fractions were isolated, and pro-caspase-1, active caspase-1, and prohibitin were measured by Western blotting. (B) Densitometry analysis of blots shown in panel A, where pro-caspase-1 and active caspase-1 levels were normalized to the loading control (prohibitin). (C) WT and *Nlrc4^−/−^* BMDMs were treated with sterile saline or inoculated with P. aeruginosa strain PA103, PA103 *exoT*::Tc, PA103 Δ*exoU exoT*::Tc, or PA103 Δ*pcrV*. At 180 min postinoculation, enriched mito-MAM fractions were isolated for Western blot analysis. (D) Densitometry analysis of blots shown in panel C, where pro-caspase-1 and active caspase-1 levels were normalized to the loading control (prohibitin). *, *P* value = 0.03. (E) WT BMDMs were preincubated with DMSO (vehicle control) or the ExoU-specific inhibitor PsA (50 μM), where compounds were added 60 min prior to inoculation. Subsequently, cultures were treated with sterile saline or inoculated with P. aeruginosa strain PA103 for 180 min (DMSO or PsA was maintained throughout the time course). Enriched mito-MAM fractions were isolated, and pro-caspase-1, active caspase-1, active IL-1β, and prohibitin were measured by Western blotting. (F) Densitometry analysis of caspase-1 (top) and IL-1β (bottom) from the blots in panel E (normalized to prohibitin). **, *P* value = 0.0014. (G) NLRC4-FLAG BMDMs were treated as described for panel A. Enriched mito-MAM fractions were isolated and assayed for NLRC4-FLAG (112 kDa), pro-caspase-1, active caspase-1, IL-1β, and prohibitin, measured by Western blotting. (H) Densitometry analysis of NLRC4-FLAG (top), caspase-1 (middle), and IL-1β (bottom) from the blots in panel G (normalized to prohibitin). *, *P* value = 0.0126. All blots and densitometry analyses are representative of 3 independent experiments.

The data presented thus far implicate associations between ExoU, mitochondria, and NLRC4 during P. aeruginosa infection. Interestingly, the NLRP3 inflammasome has been previously shown to localize to mitochondria and activate caspase-1 in response to cardiolipin (50, 56). Thus, we next asked whether NLRC4 and caspase-1 associate with mitochondria during P. aeruginosa infection. To determine this, we enriched mito-MAMs from uninfected and P. aeruginosa-inoculated BMDMs and examined caspase-1 activation (using prohibitin as the loading control). Interestingly, pro-caspase-1 was detected in enriched mitochondria and MAM fractions at 60 min postinoculation, and ExoU inhibited caspase-1 activation (Fig. 4A and B). To further extend our observations, we examined the effects of ExoU on caspase-1 activation by comparing enriched mito-MAM fractions from wild-type versus *Nlrc4^−/−^* BMDMs at 180 min postinoculation. Figure 4C and D show that caspase-1 activation detected in enriched mito-MAMs was dependent on NLRC4 and that ExoU, with or without ExoT, inhibited caspase-1 activation.

Next, we sought to determine whether ExoU PLA_2_ enzyme activity was required for the observed inhibition of caspase-1 activation at the mitochondria. Inoculations were performed with and without addition of the ExoU-specific inhibitor, PsA (55), to the culture medium (added 60 min prior to inoculation and maintained throughout the time course). Indeed, compared to the case with the vehicle-only control (DMSO), the addition of PsA reversed the inhibitory effect of ExoU on caspase-1 activation (Fig. 4E and F). As a further control, we also determined that PsA reversed the inhibitory effect of ExoU on IL-1β activation, although the data did not reach statistical significance (Fig. 4E and F). Finally, to determine whether NLRC4 also associates with mitochondria, we used BMDMs isolated from a transgenic mouse expressing NLRC4-3×Flag (57) to allow NLRC4 detection with an anti-Flag antibody (Fig. 4G and H). NLRC4-3×Flag was detected in the enriched mito-MAM fractions, and this model recapitulated ExoU-mediated inhibition of caspase-1 and IL-1β activation. Together, these data indicate that ExoU inhibits caspase-1 and IL-1β activation at the mitochondria at both early and late time points postinoculation.

## DISCUSSION

P. aeruginosa is an important cause of VAP in critically ill patients and often results in a dysregulated inflammatory response that underlies severe conditions such as ARDS and sepsis (2–5). However, beyond antibiotics and supportive care, there are no therapeutics that target the underlying causes of ARDS and sepsis. Difficulties associated with treatment of VAP, ARDS, and sepsis are further compounded by the fact that P. aeruginosa is an ESKAPE pathogen, notorious for antibiotic resistance. With limited therapeutic options to treat either the infection or the dysregulated host response, there is a need to identify novel targets for intervention. Here, we describe mechanisms by which the P. aeruginosa T3SS effector protein, ExoU, provides the pathogen with a competitive advantage by damaging host mitochondria and inhibiting the host inflammatory response.

The P. aeruginosa T3SS effector, ExoU, elicits cytolytic and noncytolytic effects on infected host cells to enhance virulence. ExoU plays a critical, pathogenic role by lysing the initial wave of immune cells infiltrating into the lung (21, 30), allowing the pathogen to persist, replicate, and disseminate from the lung. Noncytolytic effects of ExoU result in differential IL-1 family cytokine activation in the lung to augment P. aeruginosa virulence (45) and dysregulate host eicosanoid generation to exacerbate neutrophil migration and damage the lung (46). Evidence supporting the temporal nature of ExoU pathogen-host interactions comes from studies using a tetracycline-inducible system, which show that *exoU* expression within the first 3 h of infection is critical to virulence in a murine pneumonia model (23). If *exoU* transcription is induced after 3 h of infection, the bacterial strain is rendered with attenuated virulence. In addition, previous studies (43, 44) along with work presented herein (Fig. 1 and 4) demonstrated that ExoU transiently inhibits NLRC4 inflammasome-mediated inflammation early during the initial pathogen-host interaction. Our observation that ExoT is also able to inhibit NLRC4 inflammasome activation in BMDMs is different than a previous report in the literature (44). However, there are notable differences between the studies, such as the bacterial growth conditions used. Thus, future studies are required to determine the effects of bacterial growth phase and stress responses on the interactions between P. aeruginosa and immune cells.

Our studies also suggest that ExoU exerts effects on the host cell at later time points postinoculation by inducing mitochondrial oxidative stress and damage along with inhibiting caspase-1 and IL-1β activation at the mitochondria (Fig. 2 and 4). In addition, the observation that NLRC4 and caspase-1 were associated with mito-MAM fractions enriched from BMDMs suggests that the NLRC4 inflammasome assembles at the mitochondria. Previous studies have shown localization of pro-caspase-1 and activation of the NLRP3 inflammasome at the mitochondria in response to cardiolipin release (50, 56), but to our knowledge, this is the first report that NLRC4 may also associate with mitochondria to activate caspase-1. While additional studies are required to fully determine whether the NLRC4 inflammasome is activated at the mitochondria, these observations raise the tantalizing prospect that caspase-1 compartmentalization governs its function. In effect, caspase-1 released to the extracellular milieu may control inflammatory responses to infection, whereas mitochondrion-associated caspase-1 may control cellular behaviors such as metabolism and mitochondrial function (58, 59). Further studies are required to determine the nature of mitochondrion-associated caspase-1 targets.

One of the best-known aspects of ExoU in P. aeruginosa virulence is its localization to the host plasmalemmal membrane (19, 22, 60). Upon injection by the T3SS, ExoU localizes to the membrane via C-terminal residues residing between positions 550 and 687 and through binding to phosphatidylinositol 4,5-bisphosphate [PI(4,5)P_2_]. ExoU PLA_2_ is then activated through interactions with ubiquitinylated proteins and membrane inositol phosphatides (61–65). Our data obtained using a highly sensitive enzymatic assay to measure PLA_2_ activity suggest that ExoU may also associate with mitochondria during P. aeruginosa infection of BMDMs (Fig. 3). Furthermore, optimal ExoU association with mitochondria involved NLRC4, ExoT, and possibly ExoU PLA_2_ activity. While it is tempting to speculate that the ExoU-mitochondrial association is linked to ExoU-mediated inhibition of NLRC4 inflammasome activation, there are issues still outstanding. For example, ExoU-mediated inhibition of caspase-1 activation at the mitochondria involved ExoU PLA_2_ activity (Fig. 4E and F), independent of ExoT (Fig. 4C and D). In addition, ExoU triggered mitochondrial oxidative stress and mitochondrial damage independent of ExoT (Fig. 4). Together, these data indicate that maximal ExoU association with mitochondria involves bacterial (ExoT) and host (NLRC4) factors, but ExoU-mediated inhibition of caspase-1 activation is most highly dependent on ExoU PLA_2_ activity. In addition, there are questions related to the timing and sequence of events altered and/or coordinated by ExoU and whether ExoU-mediated effects on the NLRC4 inflammasome and mitochondria are functionally linked or distinct. The data suggest that ExoU differentially affects NLRC4 inflammasome activation early in infection, causing a transient delay in caspase-1 release to the culture medium, whereas the effects of ExoU can be observed in enriched mito-MAM fractions at later time points. Thus, further studies are required to fully delineate whether the timing or subcellular localization of ExoU and/or the NLRC4 inflammasome play a biological role during infection. Specifically, it will be important to determine the timing of ExoU association to plasmalemmal membranes, the mitochondria, or other intracellular membranes and the attendant effects on mitochondrial function and NLRC4 inflammasome activation during P. aeruginosa infection. Moreover, whether the other exoenzyme effectors ExoS, ExoT, and ExoY also associate with mitochondria to disrupt inflammatory cell function during infection remains an important question for future studies.

Despite being present in only 30% of P. aeruginosa clinical strains, *exoU* correlates with some of the most adverse patient outcomes (2, 28). In addition, the presence of *exoU* also correlates with P. aeruginosa strains that exhibit very high levels of antibiotic resistance (66, 67). Our studies provide further insight into the constellation of P. aeruginosa virulence traits associated with *exoU*. During P. aeruginosa infection of BMDMs, ExoU delivers at least two distinct and potentially interrelated hits to a host cell that transiently inhibit the NLRC4 inflammasome response and trigger mitochondrial damage and dysfunction. ExoU-mediated subversion of the host inflammatory response positions the pathogen for immune avoidance, proliferation, and dissemination from the initial site of infection. Thus, considering that poor patient outcomes during critical illnesses such as pneumonia, ARDS, and sepsis are defined by a dysregulated host response to infection (33, 68–70), it is critical to understand the mechanisms by which pathogen virulence factors drive host dysregulation. Studies are ongoing to examine mechanisms underlying ExoU-mediated inhibition of the NLRC4 activation during P. aeruginosa infection and to determine whether these observations translate to animal models of infection and, ultimately, to human cells. In addition, future studies are required to determine the specific interactions of ExoU with NLRC4 and/or the upstream pattern recognition receptor NAIP.

## MATERIALS AND METHODS

### Reagents.

Dulbecco’s modified Eagle medium (DMEM) was from Santa Cruz Biotech; DMEM without phenol red (cDMEM) was from Thermo Fisher. cDMEM was supplemented with 4 mM l-glutamine (Corning; glutagro supplement) and 1 mM sodium pyruvate (scDMEM). Fetal bovine serum (FBS) was from Bio-techne (formerly Atlanta Biologicals). Novex Sharp prestained protein standard was from Invitrogen. Rabbit monoclonal anti-LC3A/B and rabbit monoclonal cleaved anti-IL-1β (Asp117) primary antibodies were from Cell Signaling Technology (catalog numbers 12741S and 52718S, respectively; used at a 1:1,000 dilution). Mouse monoclonal anti-FLAG M2 antibody was from Sigma (catalog number F3165; used at a 1:50 dilution). Mouse monoclonal anti-caspase-1 antibody was from Adipogen (catalog number AG-20B-0042; used at a 1:1,000 dilution). Mouse monoclonal anti-VDAC-1 (used at a 1:200 dilution) antibody was from Santa Cruz (catalog number sc-390996). Rabbit polyclonal anti-prohibitin antibody was from Invitrogen (catalog number PA5-27329; used at a 1:500 dilution). Goat anti-mouse-horseradish peroxidase (HRP) and goat anti-rabbit-HRP secondary antibodies were from Thermo Fisher (catalog numbers 62-6520 and 31460, respectively; used at a 1:2,000 dilution). Pseudolipasin A was a custom synthesis from Chembridge (dissolved in 100% anhydrous DMSO and used at 50 μM). Super Signal West Femto was from Thermo Fisher.

### Bacterial strains and culture conditions.

The P. aeruginosa strains and plasmids used in this study were kindly provided by Dara Frank (Medical College of Wisconsin). Strains tested included wild-type PA103 encoding ExoU and ExoT, isogenic PA103 mutants encoding ExoU only (*exoT*::Tc) or ExoT only (Δ*exoU*) or lacking both ExoU and ExoT (Δ*exoU exoT*::Tc), and a T3SS mutant (Δ*pcrV*) (genotypes described in Table 1). Previous studies have shown that individual effector protein mutations in PA103 along with the complemented plasmids restore the ExoU-dependent phenotype (19, 47, 48). In addition, these previously described pUCP18 derivative plasmids were used to express wild-type ExoU or an activity-null mutant (S142A) in the PA103 Δ*exoU* and PA103 Δ*exoU exoT*::Tc mutant strains used for the studies described here. Plasmids were introduced by electroporation and transformants selected on lysogeny broth (LB) agar medium (Luria-Bertani, Lennox formulation, grown overnight at 37°C) containing carbenicillin (400 μg/mL). All cultures were maintained as frozen stock solutions (at −80°C) and routinely cultured overnight at 37°C on agar plates containing the minimal E salts medium of Vogel and Bonner (containing antibiotics as appropriate). Prior to infection experiments, bacteria were scraped into 10 mL of sterile normal saline solution (SS), collected by centrifugation (5,000 × *g*, 10 min, at room temperature), and suspended in 1 mL of SS. Spectrophotometry used to determine culture optical density at 600 nm (OD_600_). We routinely determined the correlation between OD_600_ and CFU per milliliter for bacterial strains by serial dilution and direct plate counts on LB agar medium (grown overnight at 37°C) (71).

### Cultured bone marrow-derived macrophages.

Primary murine bone marrow-derived macrophages (BMDMs) were isolated from femurs and tibias of C57BL/6J (WT) strain derivatives from The Jackson Laboratory (JAX). The generation of *Nlrc4*^−/−^, NLRC4-FLAG, and *caspase-1/11^−/−^* mice was previously described (57, 72, 73). *caspase-1/11*^−/−^ mice were purchased from JAX. Mouse breeding colonies were all maintained in an AAALAC-approved specific-pathogen-free vivarium facility, and all experimental protocols were reviewed and approved by the University of South Alabama IACUC. Cells were cultured in DMEM supplemented with 10% FBS, 10 mM HEPES (pH 7), 15% L929-conditioned medium (as a source of macrophage colony-stimulating factor), and 100 U/mL of penicillin-streptomycin at 37°C and 5% CO_2_ for the first 3 days postharvest. Subsequently, media were changed every 3 days without the addition of penicillin-streptomycin. Cells were cultured for 7 to 10 days at 37°C and 5% CO_2_ before use.

### Infection of BMDMs with P. aeruginosa.

BMDMs were seeded the day prior to infection at 6 × 10^5^ cells per well in a 12-well tissue culture plate (WT and *caspase-1/11^−/−^*) or at 2 × 10^6^ per well in a 6-well tissue culture plate (*Nlrc4^−/−^*) and grown overnight at 37°C and 5% CO_2_. The next day, medium was exchanged and BMDMs were incubated in scDMEM without serum or L929-conditioned medium at 37°C and 5% CO_2_ for 1 h before infection. Note that for this model we did not prime the macrophages with a Toll-like receptor agonist (e.g., lipopolysaccharide [LPS]) in order to better isolate the effects of the NLRC4 inflammasome from that of the NLRP3 inflammasome. As a consequence, the levels of IL-1β were very low in the early time points postinoculation. Infections were performed at a multiplicity of infection (MOI) of 20 or 40 bacteria per host cell (20:1 or 40:1) by removing culture medium and replacing it with 0.33 mL (12-well plate) or 1 mL (6-well plate) of scDMEM containing different P. aeruginosa strains at the desired density. Control macrophages were treated with sterile saline solution alone (control condition). Infected cells were incubated at 37°C and 5% CO_2_. At 60, 120, or 180 min postinoculation, the culture medium was transferred into a 2-mL microcentrifuge tube and bacteria were removed by centrifugation (21,000 × *g*, 5 min, room temperature). ExoU-expressing strains often caused cell lifting; therefore, floating cells were collected from the culture medium by a low-speed centrifugation (400 × *g*, 4 min, room temperature) before the high-speed centrifugation and combined with the monolayer in the wells. Detection of proteins in culture supernatants at early time points often required combining multiple wells. For cellular lysates, the cell monolayers were lysed in ice cold lysis buffer containing 0.5% SDS, 1% Triton X-100, 1× phosphate-buffered saline (PBS; pH 7.4), and 1× protease inhibitor cocktail (cOmplete, EDTA free; Roche). Cells were scraped into lysis buffer and transferred to 1.5-mL microcentrifuge tubes and stored at −80°C. Downstream sample processing is described below.

### Mitochondrial superoxide production.

Wild-type BMDMs were seeded at 1 × 10^6^ in a 35-mm culture dish with inset coverslip and grown overnight at 37°C and 5% CO_2_ (MatTek; part number P35G-1.5-14-C). The next day, BMDMs were preincubated with 5 μM MitoSOX Red (Thermo Fisher) in 2 mL of scDMEM for 1 h at 37°C and 5% CO_2_. BMDMs were treated with sterile saline (control condition) or infected for 3 h in scDMEM at an MOI of 20. Infection medium was removed and fresh scDMEM added to cells. As negative and positive controls, BMDMs were incubated with 2 mL of scDMEM alone and 400 μM hydrogen peroxide (H_2_O_2_) for 3 h, respectively. For each culture dish/coverslip, a series of 30 to 40 images were randomly acquired using a Nikon Eclipse TE2000-U fluorescence microscope with a 60× objective. Subsequently, 10 random images were selected and analyzed using Metamorph software to determine individual cell signal intensities. P. aeruginosa infection images are representative of 3 to 7 independent experiments. The H_2_O_2_-treated images are representative of 2 independent experiments performed in duplicate. We compiled the average intensity per image for 10 randomly selected images across each culture dish/coverslip for each independent experiment. Compiled average intensities per image were normalized to the saline control and are shown in Fig. 2.

### Enrichment of mitochondria and mito-MAM fractions.

BMDMs were seeded to confluence the day before at 3.5 × 10^7^ cells per 150-mm petri dish. BMDMs were incubated in scDMEM at 37°C and 5% CO_2_ for 1 h before infection. Infections were performed at an MOI of 40 by removing culture medium and replacing it with 10 mL of scDMEM containing different P. aeruginosa strains at the desired density. Control macrophages were treated with saline solution only (control condition). Infections were incubated at 37°C and 5% CO_2_ for 3 h. At 180 min postinoculation, cells were lifted using 6 mL of 1× Versene solution (Thermo Fisher) and incubated at 37°C for 10 min. Similar to the case described above, floating cells were collected from the culture medium by a low-speed centrifugation first (500 × *g*, 4 min, room temperature) and combined with the monolayer in the dish. Dishes were rinsed with cDMEM plus 10% FBS. Cells were centrifuged to pellet at 500 × *g* for 5 min. Pellets were suspended in 1 mL of cDMEM plus 10% FBS and then transferred to a 2-mL microcentrifuge tube. Mitochondrion-associated membrane (mito-MAM) fractions were then enriched using a mitochondrial isolation kit for cultured cells as per the manufacturer’s instructions (Thermo Fisher; catalog number 89874). To assess the efficacy of mito-MAM fraction enrichment, we compared mito-MAM fractions to cytosolic fractions using an antibody against mitochondrial proteins in a Western blot.

### Detection of caspase-1, IL-1β, LC3, and VDAC-1.

Cellular lysates were sonicated to reduce sample viscosity and diluted 1:5 to determine cellular protein concentration using the Bio-Rad DC protein assay kit (note that mitochondrial lysates were not diluted). Samples were prepared at the desired protein concentration in Laemmli loading dye containing 2.5% 2-mercaptoethanol (β-ME). A total of 20 to 30 μg for cellular lysates or 2.5 μg for mitochondrial lysates was resolved by SDS-PAGE on 4 to 12% bis-Tris gels (NuPage; Invitrogen) in 1× morpholineethanesulfonic acid (MES)-SDS running buffer (Invitrogen). Gels were run at 185 V for 65 to 75 min and then transferred to 0.2-μm nitrocellulose membranes. Gels were transferred in 1× transfer buffer (Invitrogen) with 20% methanol on ice at 100 V for 60 min (note that transfer conditions were changed to 110 V for 90 min to detect NLRC4-FLAG protein). Transfer was verified by Ponceau S staining of the nitrocellulose membranes. After blotting and image acquisition, membranes were stripped and reprobed for β-actin as a loading control for cellular lysates (1:1,000).

Active forms of caspase-1 and IL-1β were detected in cell culture supernatants by Western blotting. Culture supernatants from duplicate wells were treated with trichloroacetic acid (TCA) at 10% of final volume and incubated overnight on ice at 4°C. TCA samples were centrifuged (18,000 × *g*, 45 min, 4°C), washed with 100% ethanol, and then collected by centrifugation (18,000 × *g*, 15 min, 4°C). Pellets were allowed to dry for 10 min at 60°C and then dissolved directly in 1× Laemmli loading dye (with β-ME). All Western blot images were acquired using a Bio-Rad ChemiDoc XRS and analyzed using Quantity One software.

### ExoU PLA_2_ *in vitro* activity assay.

Enriched mito-MAM fractions were collected at defined time points postinoculation and collected by centrifugation according to the manufacturer’s instructions. The resulting pellets were directly suspended in 50 mM MOPS (morpholinepropanesulfonic acid; pH 7.4)/50 mM NaCl buffer solution containing the fluorogenic PLA_2_ substrate PED6 [*N*-((6-(2,4-dinitrophenyl)amino)hexanoyl)-2-(4,4-difluoro-5,7-dimethyl-4-bora-3a,4a-diaza-*s*-indacene-3-pentanoyl)-1-hexadecanoyl-*sn*-glycero-3-phosphoethanolamine, triethylammonium salt; Thermo Fisher; 29.7 μM] (74). Assays were performed as previously described (15, 75, 76). Briefly, reaction mixtures were incubated for 25 min to facilitate formation of enzyme-substrate complexes, followed by baseline fluorescence measurements. Then, polyubiquitin (pUb; 0.1 mg/mL) was added to activate ExoU (control reactions were without pUb). Fluorescence was measured every hour for 3 h using a NanoDrop 3300 microvolume fluorospectrometer. Excitation was at 470 nm ± 10 nm, emission was at 511 nm, and the fluorometer scan was done at 500 to 750 nm. Data points were corrected by subtracting fluorescence intensity measured in the background reaction incubated with substrate (expressed as relative fluorescent units [RFUs]). The rate of PED6 hydrolysis was expressed as RFUs per minute and normalized to the total mitochondrial protein added to the reaction. As a positive control, we used recombinant ExoU with an N-terminal His_10_ tag (N-His_10_-ExoU) purified as described previously (15). To generate an ExoU enzyme activity standard curve, we determined the RFUs per minute for reaction mixtures containing 0.05, 0.1, 0.5, and 1 μg of purified N-His_10_-ExoU. This standard curve was used to estimate the amount of ExoU detected in the enriched mito-MAM fractions (based on the average RFUs per minute detected in the presence of pUb across an average of 3 independent experiments).

### Confocal fluorescence microscopy.

Wild-type BMDMs were seeded at 1.5 × 10^5^ per well on 8-well chambered cover glass slides the day prior to an experiment. Prior to infection, cells were incubated for 1 h with MitoTracker Red CMXRos (500 nM; Invitrogen) in scDMEM at 37°C and 5% CO_2_. Cells were treated with sterile saline solution (control condition) or infected with P. aeruginosa strain PA103 or PA103 Δ*exoU exoT*::Tc at an MOI of 20 for 3 h. At experiment terminus, cells were fixed in 2.5% formalin in 1× PBS (pH 7.4) for 15 min at room temperature. Slides were washed in 1× PBS (pH 7.4) and then incubated with blocking solution (5% normal goat serum, 0.3% Triton X-100, and 1× PBS [pH 7.4]) for 1 h at room temperature. LC3A/B Alexa Fluor 488 (catalog number 13082S; Cell Signaling Technology; 1:100) primary antibody was added to designated wells overnight at 4°C protected from light. Cells were stained with Hoechst (1:1,000) the following day for 1 h at room temperature. Cells were washed in 1× PBS (pH 7.4) three times for 5 min each. Images were acquired using a Nikon A1r scanning confocal microscope with a Plan-Aprochromat 60× objective. For colocalization studies, images from 6 fields of view over 3 independent experiments were analyzed with ImageJ software using Just Another Colocalization Plugin (JACoP) (77), and data were exported for analysis.

### Statistics.

Data are reported as means ± standard s from at least 3 independent experiments. Prism 8 (GraphPad Software, San Diego, CA) was used for all analyses. For colocalization analysis, one-way analysis of variance (ANOVA followed by Tukey’s *post hoc* test was performed. For multiple-comparison analysis, two-way ANOVA followed by Tukey’s *post hoc* test was performed. For the mitochondrial oxidative stress experiments, data were determined to be nonnormally distributed by the D’Agostino and Pearson normality test and thus, all groups were compared by one-way ANOVA with Kruskal-Wallis *post hoc* test. Differences with a *P* value of <0.05 were considered significant.
