# Supplementary material for: The Pseudomonas aeruginosa Type III Secretion System Exoenzyme Effector ExoU Induces Mitochondrial Damage in a Murine Bone Marrow-Derived Macrophage Infection Model
Source: Infect Immun. 2022 Mar 17;90(3):e00470-21. doi: 10.1128/iai.00470-21 (PMC8929383; doi:10.1128/iai.00470-21)
Supplement: Supplemental file 1 — Supplemental material. Download iai.00470-21-s0001.pdf, PDF file, 0.5 MB [file iai.00470-21-s0001.pdf]

The *Pseudomonas aeruginosa* type III secretion system exoenzyme effector ExoU induces mitochondrial damage in a murine bone marrow derived macrophage infection model

Kierra S. Hardy[1,2], Amanda N. Tuckey[1,2], Nicole A. Housley[1,2], Joel Andrews[3], Mita Patel[4], Abu-Bakr Al-Mehdi[4], Robert A. Barrington[1,2], Suzanne L. Cassel[5], Fayyaz S. Sutterwala[5], and Jonathon P. Audia[1,2,6]

Department of Microbiology and Immunology[1]; Center for Lung Biology[2]; Mitchell Cancer Institute[3]; Department of Pharmcology[4]; University of South Alabama College of Medicine, Mobile, AL USA

Women's Guild Lung Institute, Department of Medicine, Cedars-Sinai Medical Center, Los Angeles, CA USA[5]

[6]Corresponding Author: Jonathon P. Audia, University of South Alabama College of Medicine, Medical Sciences Building, 5851 USA North Drive, Mobile, AL 36688, 251-460-6929, jaudia@southalabama.edu

Running title: *Pseudomonas aeruginosa* ExoU targets host mitochondria

Key words: *Pseudomonas aeruginosa*, ExoU, ExoT, NLRC4 inflammasome, caspase-1, mitochondria, pneumonia, sepsis

Supplemental Figure 1

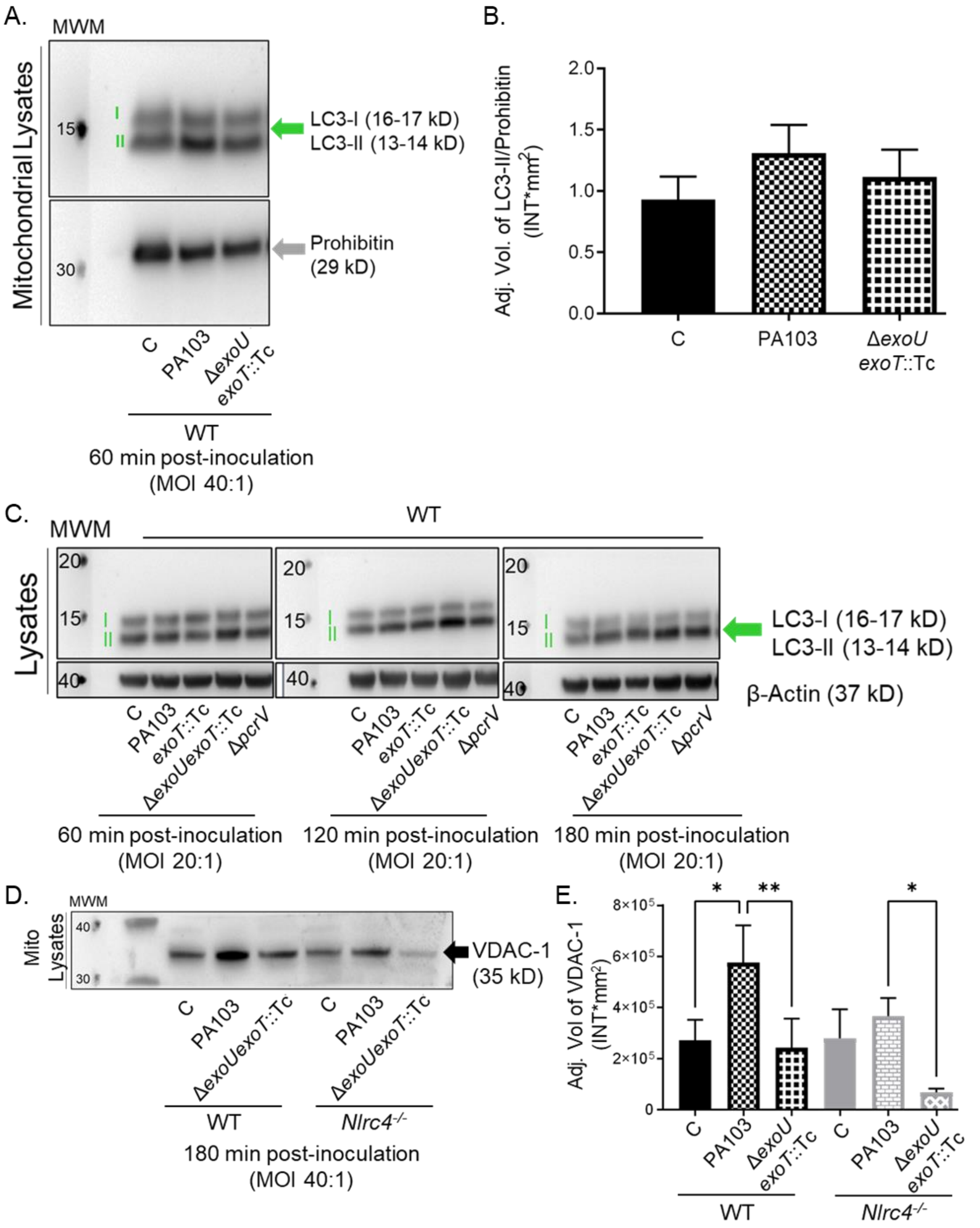

FIGURE S1. A) WT BMDMs were treated with sterile saline (C) or inoculated with *P. aeruginosa* strains PA103 or PA103 (Δ*exoU exoT*::Tc) (see **Table 1** for genotype information). At 60 minutes post-inoculation, enriched mitochondrial and MAM fractions were isolated, and LC3-I, LC3-II (green arrow), and Prohibitin (grey arrow) were measured in lysates by Western blot. **B**) Densitometry analysis of blots from panel **A** (normalized to Prohibitin). **C**) WT BMDMs were treated with sterile saline (Ctrl) or inoculated with *P. aeruginosa* strains PA103, PA103 (*exoT*::Tc), PA103 (Δ*exoU exoT*::Tc), or PA103 (Δ*pcrV*) over a time course. LC3-I and LC3-II were measured in whole cell lysates by Western blot (β-actin was used as the loading control). **D**) WT and *Nlrc4*$^{-/-}$ BMDMs were treated with sterile saline (C) or inoculated with *P. aeruginosa* strains PA103 or PA103 (Δ*exoU exoT*::Tc), and VDAC-1 (light blue arrow) measured in enriched mitochondrial and MAM extracts by Western blot. **E**) Densitometry analysis of blots from panel **D**. **p *value* = 0.0089, *p *value* < 0.017. MWM = molecular weight marker. Data are representative of 3 independent experiments.

Supplemental Table 1

Comparison of Conditions	Mean Rank Diff	Significance	*P* value
Control (Saline) vs. Δ*exoU*	7.635	ns	>0.9999
Control (Saline) vs. Δ*exoU*/pExoU	-68.92	ns	0.9204
Control (Saline) vs. Δ*exoU*/pExoU(S142A)	6.56	ns	>0.9999
Control (Saline) vs. PA103	-153.1	****	<0.0001
Control (Saline) vs. Δ*exoU exoT*::Tc	21.95	ns	>0.9999
Control (Saline) vs. *exoT*::Tc	-196.5	****	<0.0001
Control (Saline) vs. Δ*pcrV*	-48.63	ns	>0.9999
Control (Saline) vs. Δ*exoU exoT*::Tc/pExoU	-92.76	ns	0.4680
Control (Saline) vs. Δ*exoU exoT*::Tc/pExoU(S142A)	17.7	ns	>0.9999
Δ*exoU* vs. Δ*exoU*/pExoU	-76.56	*	0.0248
Δ*exoU* vs. Δ*exoU*/pExoU(S142A)	-1.075	ns	>0.9999
Δ*exoU* vs. PA103	-160.7	****	<0.0001
Δ*exoU* vs. Δ*exoU exoT*::Tc	14.32	ns	>0.9999
Δ*exoU* vs. *exoT*::Tc	-204.1	****	<0.0001
Δ*exoU* vs. Δ*pcrV*	-56.26	ns	0.8067
Δ*exoU* vs. Δ*exoU exoT*::Tc/pExoU	-100.4	*	0.0414
Δ*exoU* vs. Δ*exoU exoT*::Tc/pExoU(S142A)	10.07	ns	>0.9999
Δ*exoU*/pExoU vs. ΔU/pExoU(S142A)	75.48	*	0.0335
Δ*exoU*/pExoU vs. PA103	-84.16	*	0.0218
Δ*exoU*/pExoU vs. Δ*exoU exoT*::Tc	90.87	***	0.0010
Δ*exoU*/pExoU vs. *exoT*::Tc	-127.6	****	<0.0001
Δ*exoU*/pExoU vs. Δ*pcrV*	20.3	ns	>0.9999
Δ*exoU*/pExoU vs. Δ*exoU exoT*::Tc+ pExoU	-23.84	ns	>0.9999
Δ*exoU*/pExoU vs. Δ*exoU exoT*::Tc+ pExoU(S142A)	86.62	ns	0.1610
Δ*exoU*/pExoU(S142A) vs. PA103	-159.6	****	<0.0001
Δ*exoU*/pExoU(S142A) vs. Δ*exoU exoT*::Tc	15.39	ns	>0.9999
Δ*exoU*/pExoU(S142A) vs. *exoT*::Tc	-203.1	****	<0.0001
Δ*exoU*/pExoU(S142A) vs. Δ*pcrV*	-55.19	ns	0.9601
Δ*exoU*/pExoU(S142A) vs. Δ*exoU exoT*::Tc+ pExoU	-99.32	*	0.0499
Δ*exoU*/pExoU(S142A) vs. Δ*exoU exoT*::Tc+ pExoU(S142A)	11.14	ns	>0.9999
PA103 vs. Δ*exoU exoT*::Tc	175	****	<0.0001
PA103 vs. *exoT*::Tc	-43.42	ns	>0.9999
PA103 vs. Δ*pcrV*	104.5	**	0.0020
PA103 vs. Δ*exoU exoT*::Tc/pExoU	60.32	ns	>0.9999
PA103 vs. Δ*exoU exoT*::Tc/pExoU(S142A)	170.8	****	<0.0001
Δ*exoU exoT*::Tc vs. *exoT*::Tc	-218.5	****	<0.0001
Δ*exoU exoT*::Tc vs. Δ*pcrV*	-70.58	ns	0.0998
Δ*exoU exoT*::Tc vs. Δ*exoU exoT*::Tc/pExoU	-114.7	**	0.0052
Δ*exoU exoT*::Tc vs. Δ*exoU exoT*::Tc/pExoU(S142A)	-4.25	ns	>0.9999
exoT::Tc vs. Δ*pcrV*	147.9	****	<0.0001
exoT::Tc vs. Δ*exoU exoT*::Tc/pExoU	103.7	*	0.0444
exoT::Tc vs. Δ*exoU exoT*::Tc/pExoU(S142A)	214.2	****	<0.0001
Δ*pcrV* vs. Δ*exoU exoT*::Tc/pExoU	-44.14	ns	>0.9999
Δ*pcrV* vs. Δ*exoU exoT*::Tc/pExoU(S142A)	66.33	ns	>0.9999
Δ*exoU exoT*::Tc/pExoU vs. Δ*exoU exoT*::Tc/pExoU(S142A)	110.5	ns	0.1026
